# Association Between Passive Smoking and the Risk of Cervical Intraepithelial Neoplasia 1 in Korean Women

**DOI:** 10.2188/jea.JE20160118

**Published:** 2018-01-05

**Authors:** Kyung-Jin Min, Jae-Kwan Lee, Kyeong A So, Mi Kyung Kim

**Affiliations:** 1 Department of Obstetrics and Gynecology, College of Medicine, Korea University, Seoul, Korea; 2 Translational Epidemiology Research Branch, Division of Cancer Epidemiology and Management, National Cancer Center, Goyang, Korea; 3 Cheil General Hospital & Women’s Healthcare Center, Dankook University College of Medicine, Seoul, Korea

**Keywords:** passive smoking, smoking, cervical intraepithelial neoplasia, cervical cancer

## Abstract

**Background:**

The role of passive smoking on cervical carcinogenesis remains controversial. We investigated the association of passive smoking with the risk of cervical intraepithelial neoplasia (CIN) and cervical cancer.

**Methods:**

The study recruited 1,322 women, aged 18–65 with normal cytology (*n* = 592), CIN1 (*n* = 420), CIN2/3 (*n* = 165), and cervical cancer (*n* = 145) from 2006 to 2009. This study is a cross-sectional analysis using the baseline data from the Korean human papillomavirus (HPV) cohort study. Detailed information on smoking behaviors and lifestyles were collected using questionnaires. Multinomial logistic regression analysis was performed to estimate multivariable-adjusted odds ratios (ORs).

**Results:**

Passive smoking was not statistically related to the risk of CINs and cervical cancer. However, passive smoking among non-smokers was associated with higher CIN 1 risk (OR 1.53; 95% confidence interval [CI], 1.07–2.18), compared to not passive smoking, after adjusting for demographic factors, lifestyles, and oncogenic-HPV infection status. CIN 1 risk increased with longer time exposed to passive smoking (*P* for trend <0.0003). Multivariate odds of <2 hours/day of passive smoking and that of ≥2 hours/day of passive smoking were 2.48 (95% CI, 1.49–4.14) and 2.28 (95% CI, 1.21–4.26) for CIN 1, compared to not passive smoking.

**Conclusions:**

This study found that passive smoking among non-smoking women is associated with the risk of CIN 1.

## INTRODUCTION

Cervical cancer is the third most common cancer among women worldwide, with an estimated 527,600 cervical cancer cases and 265,700 deaths occurring in 2012 worldwide.^1^ Infection with human papillomavirus (HPV) is the most important causative factor for cervical intraepithelial neoplasia (CIN) or cervical cancer. The natural progression of cervical carcinogenesis is thought to begin with an initial infection of the metaplastic epithelium in the cervical transformation zone by oncogenic-type HPV.^2^ However, most HPV infections disappear spontaneously within 2–4 years, and only a small percentage progress to low- or high-grade CINs. Therefore, other factors, in addition to HPV infection, are thought to promote the progression from infection to malignant disease.

Cigarette smoking is established as a cofactor of HPV for cervical cancer.^3^ The prevalence of HPV is associated with active smoking, especially smoking intensity.^4^ The association with smoking, which seems to be dose-dependent and disappears after smoking cessation, was confirmed in studies that restricted the analysis to HPV-positive women.^5^ Two studies have found significantly increased risks of high-grade squamous intraepithelial lesions (HSIL) and cervical cancer among HPV-positive women,^5^^,^^6^ but Syrjänen et al^7^ claimed that smoking is an independent risk factor for oncogenic HPV infection, but not for high-grade CIN.

Many studies assessing the relationship between smoking and cervical cancer have focused on active smoking only. One study, based on a pooled analysis of couples from the International Agency for Research on Cancer (IARC) multicenter case-control studies, suggested that passive smoking could not be an independent risk factor for invasive cervical cancer in the absence of active smoking.^8^ In contrast, other studies have shown a potential impact of passive smoking on the risk of cervical neoplasia.^8^^–^^12^ Other Asian countries with similar tobacco use patterns to South Korea have shown the potential effect of passive smoking on the risk of cervical neoplasia.^11^^,^^13^^,^^14^ Although there have been no reports of the association between passive smoking and development of cervical cancer in Korean women, several studies have shown that an exposure to passive smoking was positively associated with incidence of lung cancer in non-smoking women whose husbands smoke^15^ and with osteoporosis in postmenopausal never-smoking women.^16^ During 2011–2012, about 58 million nonsmokers (1 in 4 nonsmokers in the United States) were exposed to passive smoking.^17^ The Centers for Disease Control and Prevention have warned explicitly that there is no risk-free level of passive smoke exposure; even brief exposure can be harmful to health.^18^

In this study, we investigated the possible associations of active and passive smoking with the risk of cervical intraepithelial neoplasia and cervical cancer.

## MATERIALS AND METHODS

### Participant recruitment

The study recruited 1,322 women, aged 18–65 years, from March 2006 through December 2009. The study was approved by the ethics committees of NCC (NCCNCS-06-062) and of each study center. All study participants provided informed consent. Details regarding the design of the baseline measure of the HPV cohort have been described previously.^19^^–^^22^ Participants were asked to participate in this study if they met the following enrollment requirements: having sexually activity or using birth control, not being currently pregnant, having an intact uterus, having no current referral for hysterectomy, and having no history of treatment for cervical neoplasia lesions within the previous 18 months. If participants had a history of gynecological cancers; insufficient data on the questionnaire; inadequate blood for evaluation; a chronic disease, such as liver cirrhosis, renal failure, or cardiovascular disease; a drug dependency; or psychological problems, they were excluded from this study. At the time of enrolment, participants completed a questionnaire on the risk factors of cervical cancer, underwent physical and gynecological examinations, and were examined for oncogenic HPV DNA test using the Hybrid Capture 2 (HC2) (Digene Diagnostics Inc., Gaithersburg, MD, USA) and Papanicolaou smear test. Colposcopic examination and histological verification were performed at baseline with a diagnosis of atypical squamous cells of undetermined significance (ASCUS), atypical squamous cells excluding high-grade lesions (ASC-H), low-grade squamous intraepithelial lesions (LSIL), or high-grade squamous intraepithelial lesions (HSIL). In this study, we used the questionnaire results that included passive smoking history at enrollment time as an independent variable and the multiclass pathological category at enrollment time, such as normal, CIN 1, CIN 2/3, or cervical cancer, as a dependent variable.

### Pap smear test, HPV DNA test, and pathological examination

Cervical swab samples were obtained using a cervix brush (Rovers Medical Devices, Oss, Netherlands). The brush was immediately rinsed in a vial of PreservCyt solution (Cytyc Corporation, Marlborough, MA, USA), and the vial was placed in a ThinPrep Processor (Cytyc Corporation). The grade for the cytological diagnosis was based on the Bethesda classification system^23^ for Pap smear reports.

HPV DNA detection was performed using the HC2. This method is a signal-amplified hybridization antibody capture assay that utilizes chemiluminescent detection with a HPV RNA assay probe cocktail for 13 oncogenic HPV types (HPV 16, 18, 31, 33, 35, 39, 45, 51, 52, 56, 58, 59, and 68). All relative light units (RLU) measured on a luminometer were divided by the RLU of the appropriate positive control (PC) to provide a ratio. The sample was classified as a positive when the RLU/PC ratio (RLU of specimen/mean RLU of three controls) was 1 pg/mL or greater.

The histological diagnosis of cervical pathology was classified according to the following method.^24^ In cervical squamous intraepithelial lesions (SILs), the number of Ki-67-positive cells increased as the cell grading went from normal to LSIL to HSIL.^25^ Thus, the diagnostic level of Ki-67 immunostaining to detect moderate or severe dysplasia (CIN 2 or 3) was used as the cutoff value of more than 33% of Ki-67-positive nuclei in the middle and upper third layers of the epithelium.^26^

### Data collection

After the clinical visit, the survey on lifestyles and sociodemographic characteristics was performed via a personal interview at the Outpatient Department of Obstetrics and Gynecology. A wide range of information, including body size, reproductive and menstrual history, exogenous hormone use, medical history, and family history of cervical and other cancers at the study enrollment and before the onset of disease, was collected for control and case participants. The survey included final education level, occupation type, cigarette smoking, alcohol consumption, and physical activity, with a detailed time frame of exposures. Medical and laboratory data were collected, recorded, and merged into the epidemiological dataset. Medical chart was checked to insure that control participants had no history of any cancer or precancerous lesions. Details regarding the questionnaire of the HPV cohort have been described previously.^20^ Questions on smoking habits included status (never, ex-, or current smoker), intensity (number of packs smoked per day), and exposure to passive smoke (status and duration). Ex-smokers were defined as women who had abstained from tobacco smoking prior to study entry. To assess a dose-dependent relationship between active or passive smoking and CIN risk, smoking behaviors were classified according to smoking intensity (never, 1–9 packs per year, and ≥10 packs per year) and exposure time of passive smoking in household or working place (never, less than 2 hours per day, and more than 2 hours per day). The question used in the questionnaire is as follows: ‘Have you ever been exposed to passive smoking?’, ‘If you were exposed to passive smoking in your household, what was the daily average exposure time to passive smoking in the household?’, and ‘If you were exposed to passive smoking in your working place, what is the daily average exposure time to passive smoking in the working place?’.

### Statistical analysis

The χ2 test and ANOVA were used for the analysis of differences in the distribution of categorical and continuous variables, respectively. Multinomial logistic regression models were used to evaluate the association between the independent variable, passive smoking, and the categorical dependent variables, normal, CIN 1, CIN 2/3 and cervical cancer. Odds ratios (ORs) and corresponding 95% confidence intervals (CIs) were estimated, after adjustment for age, parity, oral contraceptive use, and HPV infection status. Linear trends were calculated using the median values for each risk factor as continuous variables. All analyses were conducted using SAS (SAS Institute, Cary, NC, USA).

## RESULTS

### General characteristics of study participants

The patient group with cervical dysplasia (CIN 1 and CIN 2/3) tended to be younger than the participant group with normal cytology (Table 1). Most women did not smoke tobacco or use oral contraceptives. In participants who smoked, a significant association was observed between the normal group and those with cervical CIN 1, CIN 2/3, and cervical cancer. Other demographic variables associated with the normal group and CIN 1, CIN 2/3, and cervical cancer groups (*N* = 592, 420, 165, and 145, respectively) are shown in Table 1.

**Table 1.** Selected characteristics and smoking habits of study participants

General characteristics	Normal	CIN 1	CIN 2/3	Cervical cancer	*P*^a^
	(*n* = 592)	(*n* = 420)	(*n* = 165)	(*n* = 145)	
Age, mean (SD)	45.1 (10.3)	39.9 (11.1)	40.5 (10.4)	51.2 (11.6)	<0.01
≤29, no (%)	36 (6)	87 (20)	29 (18)	3 (2)	
30–39	148 (25)	132 (32)	53 (32)	20 (14)	
40–49	195 (33)	118 (28)	53 (32)	50 (34)	
50–59	160 (27)	65 (16)	21 (13)	35 (24)	
≥60	53 (9)	18 (4)	9 (5)	37 (26)	<0.01
Cigarette smoking, no (%)					
Non-smoker	529 (89)	345 (82)	136 (82)	129 (89)	
Ex-smoker	22 (4)	32 (8)	10 (6)	8 (6)	
Current smoker	41 (7)	43 (10)	19 (12)	8 (6)	0.016
Pack-years, no (%)					
Non-smoker	529 (89)	345 (83)	136 (83)	129 (89)	
<10	50 (8)	63 (15)	22 (13)	10 (7)	
≥10	13 (2)	12 (3)	7 (4)	6 (4)	0.013
Passive smoking, no (%)					
No	366 (62)	223 (53)	84 (51)	84 (58)	
Yes	226 (38)	197 (47)	81 (49)	61 (42)	0.013
Exposure time of passive smoking, no (%)					
No	366 (81)	223 (62)	83 (80)	84 (74)	
<2 hours/day	52 (12)	89 (25)	9 (9)	20 (18)	
≥2 hours/day	33 (7)	49 (14)	12 (12)	9 (8)	<0.01
Ever use oral contraceptive, no (%)					
Never	510 (86)	349 (83)	129 (78)	119 (83)	
Current/Former	82 (14)	71 (17)	36 (22)	26 (17)	0.090
Menopausal status, no (%)					
Premenopausal	371 (63)	334 (80)	135 (82)	64 (44)	
Postmenopausal	221 (37)	86 (20)	30 (18)	81 (56)	<0.01
Number of childbirth, no (%)					
0	84 (14)	121 (29)	38 (23)	6 (4)	
1	79 (13)	66 (16)	18 (11)	11 (8)	
2	306 (52)	170 (41)	78 (47)	72 (50)	
≥3	123 (21)	63 (15)	31 (19)	56 (39)	<0.01
HPV infection status, no (%)^b^					
Negative	219 (56)	90 (24)	37 (34)	0 (0)	
Positive	170 (44)	280 (76)	73 (66)	54 (100)	<0.01

CIN, low-grade cervical intraepithelial neoplasia; SD, standard deviation.

^a^ANOVA for continuous variable and χ^2^ for categorical variable.

^b^HPV infection status was determined by detection for 13 oncogenic HPV DNA types using hybrid capture 2.

### Association between smoking or passive smoking and CINs or cervical cancer risks

Table 2 describes the association between smoking and passive smoking and the risk of CIN 1, CIN 2/3, and cervical cancer in total participants, adjusting for covariates. Smokers had significantly increasing risks of CIN 1 (OR, 1.81; 95% CI, 1.26–2.60) and CIN 2/3 (OR, 1.77; 95% CI, 1.10–2.86) in univariate multinomial logistic regressions. And passive smoking also had significantly increasing risks of CIN 1 (OR, 1.43; 95% CI, 1.11–1.84) and CIN 2/3 (OR, 1.57; 95% CI, 1.10–2.22) in univariate multinomial logistic regressions. However, after adjustment for age, parity, use of oral contraceptives, menopausal status, oncogenic-HPV infection status and smoking status, the associations between passive smoking and the risk of CIN 1, CIN 2/3 were not significant (1.26; 0.87–1.82). Smoking also were not significant with the risk of CIN 1 (1.08; 0.64–1.84) after adjusting for demographic factors, lifestyles and oncogenic-HPV infection status and passive smoking status.

**Table 2.** Association of smoking and passive smoking with the risk of CINs and cervical cancer

	Normal	CIN 1			CIN 2/3			Cervical cancer		
			
	*N* (%)^a^	*N* (%)	Univariable	Multivariable	*N* (%)	Univariable	Multivariable	*N* (%)	Univariable	Multivariable
			OR (95% CI)^b^	OR (95% CI)^c^		OR (95% CI)	OR (95% CI)		OR (95% CI)	OR (95% CI)
**Smoking status**										
Non-smoker	529 (89)	345 (82)	1 (ref.)	1 (ref.)	136 (82)	1 (ref.)	1 (ref.)	129 (89)	1 (ref.)	1 (ref.)
Smoker	63 (11)	75 (18)	1.81 (1.26–2.60)	1.08 (0.64–1.84)	29 (18)	1.77 (1.10–2.86)	0.94 (0.48–1.86)	16 (11)	1.03 (0.58–1.85)	1.67 (0.50–5.53)

**Pack-year**										
Non smoker	529 (89)	345 (82)	1 (ref.)	1 (ref.)	136 (83)	1 (ref.)	1 (ref.)	129 (89)	1 (ref.)	1 (ref.)
<10	50 (9)	63 (15)	1.89 (1.27–2.82)	1.19 (0.68–2.11)	22 (13)	1.70 (0.99–2.91)	0.86 (0.41–1.81)	10 (7)	0.82 (0.40–1.65)	1.91 (050–7.39)
≥10	13 (2)	12 (3)	1.27 (0.56–2.86)	0.58 (0.17–2.04)	7 (4)	1.75 (0.65–4.68)	1.23 (0.33–4.51)	6 (4)	1.84 (0.69–4.94)	1.15 (0.11–11.6)
*P* for linear trend			0.0109	0.9023		0.0368	0.9710		0.5528	0.5156

**Passive smoking status**										
No	366 (62)	223 (53)	1 (ref.)	1 (ref.)	84 (51)	1 (ref.)	1 (ref.)	84 (58)	1 (ref.)	1 (ref.)
Yes	226 (38)	197 (47)	1.43 (1.11–1.84)	1.26 (0.87–1.82)	81 (49)	1.57 (1.10–2.22)	1.28 (0.81–2.03)	61 (42)	1.18 (0.81–1.71)	1.12 (0.55–2.29)

**Exposure time of passive smoking**										
No	366 (81)	223 (62)	1 (ref.)	1 (ref.)	83 (79)	1 (ref.)	1 (ref.)	84 (74)	1 (ref.)	1 (ref.)
<2 hours/day	52 (12)	89 (25)	2.91 (1.98–4.28)	2.29 (1.39–3.76)	9 (9)	0.79 (0.37–1.67)	0.46 (0.19–1.09)	20 (18)	1.74 (0.98–3.07)	1.21 (0.35–4.26)
≥2 hours/day	33 (7)	49 (14)	2.38 (1.48–3.81)	1.37 (0.72–2.61)	12 (12)	1.56 (0.78–3.16)	1.09 (0.48–2.49)	9 (8)	1.16 (0.53–2.51)	0.47 (0.09–2.34)
*P* for linear trend			<0.0001	0.0711		0.3772	0.9586		0.2245	0.4464

CI, confidence interval; CIN, low-grade cervical intraepithelial neoplasia; OR, odds ratio.

^a^This number indicates that the number of participants and their percentage.

^b^Multinomial logistic regression analysis was performed to assess the association between smoking behaviors and the risk of cervical dysplasia (CIN 1, CIN 2/3, and cervical cancer).

^c^The multinomial logistic regression analysis was adjusted for age, parity, oral contraceptive use, menopausal status, oncogenic-HPV infection status and smoking status to assess the effect of passive smoking or passive smoking status to assess the effect of smoking status.

### Association between passive smoking and CINs or cervical cancer risks in nonsmokers

We performed a subgroup analysis to assess whether smoking status modify the association between passive smoking and the risk of CIN 1, CIN 2/3, and cervical cancer (Table 3). There was no significant interaction between passive smokers and smokers or oncogenic HPV infection (eTable 1 and eTable 2). In non-smokers, passive smoking had a higher CIN 1 risk (OR 1.53; 95% CI, 1.07–2.18) compared to non-passive smokers, after adjusting for demographic factors, lifestyles and oncogenic-HPV infection status. CIN 1 risk increased with longer time exposed to passive smoking (*P* for trend <0.0003). In non-smokers, women who were exposed to passive smoking for less than 2 hour per day (multivariable OR, 2.48; 95% CI, 1.49–4.14) and for 2 or more hours per day (multivariable OR 2.28; 95% CI, 1.21–4.26) showed significant associations in the risk of CIN 1. In smokers, passive smoking was not associated with the risk of CIN 1, CIN 2/3, or cervical cancer.

**Table 3.** Association of passive smoking with the risk of CINs and cervical cancer according to smoking habits of study participants

	Normal	CIN 1			CIN 2/3			Cervical cancer		
			
	*N* (%)^a^	*N* (%)	Univariable	Multivariable	*N* (%)	Univariable	Multivariable	*N* (%)	Univariable	Multivariable
			OR (95% CI)^b^	OR (95% CI)^c^		OR (95% CI)	OR (95% CI)		OR (95% CI)	OR (95% CI)
**Non-smokers^d^**										
** Passive smoking**										
No	345 (65)	193 (56)	1 (ref.)	1 (ref.)	72 (53)	1 (ref.)	1 (ref.)	80 (62)	1 (ref.)	1 (ref.)
Yes	184 (35)	152 (44)	1.47 (1.11–1.94)	1.53 (1.07–2.18)	64 (47)	1.67 (1.14–2.45)	1.52 (0.93–2.48)	49 (38)	1.15 (0.77–1.71)	1.11 (0.54–2.27)

**Exposure time of passive smoking**										
No	345 (84)	193 (65)	1 (ref.)	1 (ref.)	71 (84)	1 (ref.)	1 (ref.)	80 (78)	1 (ref.)	1 (ref.)
<2 hours/day	41 (10)	68 (23)	3.12 (2.02–4.81)	2.48 (1.49–4.14)	7 (8)	1.33 (0.55–3.18)	1.32 (0.50–3.52)	15 (15)	1.18 (0.49–2.82)	0.89 (0.18–4.31)
≥2 hours/day	25 (6)	37 (12)	2.58 (1.51–4.41)	2.28 (1.21–4.26)	7 (8)	0.87 (0.37–2.03)	0.78 (0.30–2.00)	7 (7)	1.66 (0.87–3.16)	0.36 (0.05–2.91)
*P* for linear trend			<0.0001	0.0003		0.6741	0.8162		0.286	0.5985

**Smokers**										
** Passive smoking**										
No	21 (33)	30 (40)	1 (ref.)	1 (ref.)	12 (41)	1 (ref.)	1 (ref.)	4 (25)	1 (ref.)	1 (ref.)
Yes	42 (67)	45 (60)	0.77 (0.38–1.55)	0.62 (0.26–1.49)	17 (59)	0.73 (0.29–1.80)	0.48 (0.14–1.58)	12 (75)	1.54 (0.44–5.35)	0.86 (0.10–7.47)

**Exposure time of passive smoking**										
No	21 (53)	30 (48)	1 (ref.)	1 (ref.)	12 (63)	1 (ref.)	1 (ref.)	4 (36)	1 (ref.)	1 (ref.)
<2 hours/day	11 (28)	21 (33)	1.34 (0.53–3.35)	0.90 (0.30–2.73)	2 (11)	1.09 (0.29–4.11)	0.38 (0.06–2.34)	5 (45)	1.31 (0.20–8.62)	—
≥2 hours/day	8 (20)	12 (19)	1.05 (0.37–3.01)	0.48 (0.12–1.88)	5 (26)	0.32 (0.06–1.68)	0.15 (0.01–1.52)	2 (18)	2.39 (0.53–10.7)	2.52 (0.19–33.3)
*P* for linear trend			0.8045	0.3021		0.8402	0.1894		0.5938	0.7451

CI, confidence interval; CIN, low-grade cervical intraepithelial neoplasia; OR, odds ratio.

Non-smokers indicate women who never smoked for their life but exposed or non-exposed to passive smoking.

^a^This number indicates that the number of participants and their percentage.

^b^Multinomial logistic regression analysis was performed to assess the association between smoking behaviors and the risk of cervical dysplasia (CIN 1, CIN 2/3, and cervical cancer).

^c^The multinomial logistic regression analysis was adjusted for age, parity, oral contraceptive use, menopausal status, and oncogenic-HPV infection status.

^d^Non-smokers indicate women who never smoked for their life but exposed or non-exposed to passive smoking.

## DISCUSSION

This study found that passive smoking among non-smokers is associated with the risk of CIN 1. Especially, non-smoking women who exposed to passive smoking for 2 or more hours per day (multivariable OR 2.34; 95% CI, 1.41–3.90) had higher risk of CIN 1.

Passive smoking is secondhand tobacco smoke composed of aged, exhaled mainstream smoke and diluted sidestream smoke. Sidestream smoke contains known carcinogens with similar ingredients but has a different composition than exhaled mainstream smoke, such as benzo[α]pyrene, which could potentially have a direct transformative effect on the cervix or could cause immunosuppression, allowing HPV infections to persist and progress to pre-invasive lesions and invasive cancer.^27^ Benzo[α]pyrene diol epoxide, the ultimate mutagen of benzo[α]pyrene, specifically targets the protective p53 gene, a transcription factor that regulates the cell cycle and hence functions as a tumor suppressor.^28^ Actually, nicotine and other components of tobacco smoke have been found in cervical mucus in the cervical epithelium of non-smokers.^9^ Direct cervical contact with mutagenic semen of active smoking partners could also be a plausible source of an exposure in secondhand smokers.^29^^,^^30^

Several previous studies have found that active smokers have an increased risk for cervical neoplasia.^9^^,^^13^^,^^31^^–^^34^ The four studies were unable to adjust for HR-HPV positivity to control for potential confounding factors.^31^^–^^34^ Coker et al^9^ did find that passive smoking may be associated with LSIL and HSIL, particularly if women are also HR-HPV-positive. Tsai et al^13^ asserted that, in addition to HPV infection and active cigarette smoking, exposure to passive smoking is a major risk factor for CIN. These findings are similar to our findings. However, our study is more influential because we investigated the role of passive smoking according to histological grade in cervical carcinogenesis in a large number of women. Louie et al found that passive smoking was not an independent risk factor for invasive cervical cancer in the absence of active smoking.^8^ However, their study included only CIN 3/a carcinoma in situ (CIS) as pre-invasive disease. Our study design, which separated and considered each CIN stage individually, may be better suited for resolving the role of passive smoking in cervical carcinogenesis.

Cigarette smoking among Korean women is rare (female smokers aged ≥19 years: 6.8% in 2011), but the percentage of Korean men who smoke is high (male smokers aged ≥19 years: 47.3% in 2011).^35^ The proportion of women exposed to secondhand smoke (SHS) at home (16.7% in 2011) and in the workplace (37.2% in 2011) were higher than that of active woman smokers.^35^ Although there have been no reports of the association between cervical neoplasia and SHS in Korean women, several studies show a potential influence of an exposure to SHS on the onset of other diseases.^15^^,^^16^^,^^36^ Approximately 54% of Korean women who never smoked have husbands who smoke, and the women of husbands who smoked for 30 years or more were twice as likely to develop lung cancer as women married to never-smokers.^15^ Postmenopausal Korean women whose family members were currently smokers had a higher risk (OR 3.68) of femoral neck osteoporosis compared to those with no SHS exposure.^16^ Additionally, in a population-based cohort in Korea, participants with exposure to environmental tobacco smoke had a higher risk of type 2 diabetes (hazard ratio 1.41) compared to those without such an exposure.^36^

This study showed that passive smoking is associated only with CIN 1, but not with CIN 2/3 or cervical cancer. Cervical cancer is developed via a series of multiple steps. The risk factor profiles and HPV genotype distributions in each CIN are different.^37^ In addition, according to our previous studies, fruit and vegetable consumption, mild obesity, alcohol consumption, and interaction between oral contraceptive use and active smoking are related to the different CIN stages.^19^^,^^20^^,^^22^^,^^38^^–^^40^ Several studies demonstrated the association with passive smoking in high grade cervical SIL^9^ or invasive cervical cancer.^8^ However, a larger and randomized clinical trial in 4403 predominantly Hispanic subjects reported that the time of passive smoke exposure was associated with low-grade SIL.^12^ The Japan HPV And Cervical Cancer (JHACC) study group also demonstrated that passive smoking since childhood reduced the probability of LSIL spontaneous regression within 2 years in young women.^41^ Although it is difficult to explain the reason or the mechanism for the CIN stage-specific significant association with passive smoking, passive smoking could be a critical factor in mild cervical neoplasia lesions rather than other major factors for cervical cancer.

The relationship between duration of exposure to smoking and HPV is important in the definition of exposure and cervical carcinogenesis. More passive smoking and a short period of HPV infection may not affect the occurrence of CINs, as in the inverse case. We were able to define and calculate exposure to smoking using our detailed questionnaire. However, the duration of HPV infection is difficult to know precisely. This was a limitation of our cross-sectional study. Additionally, even though we used a detailed and well-structured questionnaire written by expert surveyors, the questionnaire may have its own limitation of recall bias. Another limitation is that this study lacked data about the exact levels of nicotine in the cervix or body; therefore, additional studies are needed to obtain these data to complement our epidemiological findings. Last, the results are based on hospital patients and may not be representative of the general population due to selection bias.

In conclusion, we found that passive smoking among non-smoking women is associated with the risk of CIN 1. Further studies are needed to confirm the causal effects of passive smoking on the development cervical neoplasia, and to identify the underlying biological mechanisms.

## APPENDIX A. SUPPLEMENTARY DATA

eTable 1. The effect of interaction of smoking and passive smoking 1 on CIN risk

eTable 2. The effect of interaction of passive smoking and oncogenic HPV infection 7 on CIN risk
