# Supplementary material for: Association Between Passive Smoking and the Risk of Cervical Intraepithelial Neoplasia 1 in Korean Women
Source: J Epidemiol. 2018 Jan 5;28(1):48–53. doi: 10.2188/jea.JE20160118 (PMC5742379; doi:10.2188/jea.JE20160118)
Supplement: Supplementary file 1 [file je-28-048-s001.pdf]

eTable 1. The effect of interaction of smoking and passive smoking on CIN risk

	CIN1		CIN 2/3		Cervical cancer	
	Univariable OR (95% CI)	Multivariable OR (95% CI)	Univariable OR (95% CI)	Multivariable OR (95% CI)	Univariable OR (95% CI)	Multivariable OR (95% CI)
**Passive smoking**	1.34 (0.98–1.83)	1.38 (0.93–2.05)	1.67 (1.14–2.45)	1.44 (0.88–2.38)	1.15 (0.77–1.71)	1.11 (0.52–2.37)
**Smoking**	2.33 (1.24–4.41)	1.60 (0.70–3.65)	2.67 (1.26–5.67)	1.45 (0.55–3.83)	0.80 (0.27–2.40)	1.65 (0.28–9.75)
**Smoking + Passive smoking**	1.89 (1.14–3.09)	1.16 (0.61–2.22)	1.94 (1.04–3.59)	0.98 (0.41–2.35)	1.23 (0.62–2.45)	1.90 (0.43–8.40)
P for interaction term	0.221	0.230	0.097	0.235	0.665	0.235

CI, confidence interval; CIN, low-grade cervical intraepithelial neoplasia; OR, odds ratio.

The logistic regression analysis was adjusted for age, parity, oral contraceptive use, menopausal status, and oncogenic-HPV infection status.

Interaction term = Passive smoking status * smoking status

eTable 2. The effect of interaction of passive smoking and oncogenic HPV infection on CIN risk

	CIN 1		CIN 2/3		Cervical cancer	
	Univariable OR (95% CI)[b]	Multivariable OR (95% CI)[c]	Univariable OR (95% CI)[b]	Multivariable OR (95% CI)[c]	Univariable OR (95% CI)[b]	Multivariable OR (95% CI)[c]
**Passive smoking**	1.37 (0.83–2.26)	1.23 (0.73–2.07)	0.90 (0.51–1.59)	0.79 (0.44–1.43)	–	–
**HPV infection**	4.72 (3.26–6.82)	4.94 (3.37–7.24)	1.64 (1.05–2.56)	1.69 (1.07–2.68)	–	–
**Passive + HPV**	6.93 (4.57–10.5)	5.75 (3.72–8.89)	2.58 (1.58–4.23)	2.20 (1.31–3.68)		
P for interaction term	0.259	0.746	0.301	0.786		

CI, confidence interval; CIN, low-grade cervical intraepithelial neoplasia; OR, odds ratio.

The logistic regression analysis was adjusted for age, parity, oral contraceptive use, menopausal status, and smoking status.

Interaction term = Passive smoking status * Oncogenic-HPV infection status
